# From Gasotransmitter to Immunomodulator: The Emerging Role of Hydrogen Sulfide in Macrophage Biology

**DOI:** 10.3390/antiox12040935

**Published:** 2023-04-15

**Authors:** Alex Cornwell, Alireza Badiei

**Affiliations:** 1 Department of Biology and Wildlife, University of Alaska Fairbanks, Fairbanks, AK 99775, USA; acornwell2@alaska.edu; 2 Department of Veterinary Medicine, University of Alaska Fairbanks, Fairbanks, AK 99775, USA

**Keywords:** hydrogen sulfide, macrophage, inflammation, cellular metabolism, redox

## Abstract

Hydrogen sulfide (H_2_S) has been increasingly recognized as a crucial inflammatory mediator in immune cells, particularly macrophages, due to its direct and indirect effects on cellular signaling, redox homeostasis, and energy metabolism. The intricate regulation of endogenous H_2_S production and metabolism involves the coordination of transsulfuration pathway (TSP) enzymes and sulfide oxidizing enzymes, with TSP’s role at the intersection of the methionine pathway and glutathione synthesis reactions. Additionally, H_2_S oxidation mediated by sulfide quinone oxidoreductase (SQR) in mammalian cells may partially control cellular concentrations of this gasotransmitter to induce signaling. H_2_S is hypothesized to signal through the posttranslational modification known as persulfidation, with recent research highlighting the significance of reactive polysulfides, a derivative of sulfide metabolism. Overall, sulfides have been identified as having promising therapeutic potential to alleviate proinflammatory macrophage phenotypes, which are linked to the exacerbation of disease outcomes in various inflammatory conditions. H_2_S is now acknowledged to have a significant influence on cellular energy metabolism by affecting the redox environment, gene expression, and transcription factor activity, resulting in changes to both mitochondrial and cytosolic energy metabolism processes. This review covers recent discoveries pertaining to the involvement of H_2_S in macrophage cellular energy metabolism and redox regulation, and the potential implications for the inflammatory response of these cells in the broader framework of inflammatory diseases.

## 1. Introduction

Macrophages are cells in the innate immune system that provide the first line of defense against invading pathogens. Macrophages are classified broadly within two types of polarization states: classically activated (M1) and alternatively activated (M2) [1]. Overall, M1 macrophages are effector cells that are induced by interferon (IFN)γ and lipopolysaccharide (LPS), produce pro-inflammatory cytokines and chemokines, as well as reactive oxygen and nitrogen intermediates, and play a role in host defense against pathogens [2,3,4]. Conversely, M2 macrophages are induced by cytokines, such as IL-4 and IL-10, and play a role in tissue repair, immune regulation, and homeostasis [5]. Recent studies have revealed that hydrogen sulfide (H_2_S) acts as a potent inflammatory mediator in macrophages, modulating various activities, including migration, phagocytosis, and cytokine production, suggesting it as a promising therapeutic target for macrophage-mediated inflammatory conditions [6,7].

Gasotransmitters are a class of gaseous signaling molecules, including nitric oxide (NO), carbon monoxide (CO), and H_2_S. These gases are produced by specific enzymes in cells and serve important functional roles in regulating physiological processes, such as blood flow, inflammation, and cellular respiration [8]. Gasotransmitters differ from traditional signaling molecules, such as hormones and neurotransmitters, in that they freely diffuse through cell membranes to transduce signaling responses directly and indirectly [9]. NO is a potent signaling molecule produced by iNOS that has bactericidal and cytotoxic properties and regulates pro-inflammatory cytokine production and oxidative stress in macrophages. NO has a dual role in cellular physiology, modulating cellular metabolism and acting as a signaling molecule. NO interacts with metal-containing proteins and the electron transport chain, altering cellular respiration and regulating energy production [10]. CO is produced by the action of heme oxygenases (HO-1-3), and its anti-inflammatory role is suggested in macrophages. HO-1 is an enzyme that catalyzes the degradation of heme into biliverdin, iron, and CO [11]. The mechanisms by which gasotransmitters affect macrophage inflammation are not fully understood, yet their therapeutic potential is increasingly recognized.

H_2_S is the latest described gasotransmitter and will be the primary focus of this review. H_2_S, initially identified as an environmental toxin, is now recognized to act as an autocrine signaling molecule that was discovered to modulate an increasing number of physiological effects [6,12]. H_2_S is synthesized in mammals via de-sulfuration reactions catalyzed by the enzymes 3-mercaptosulfurtransferase (3-MST), cystathionine β-synthase (CBS), and cystathionine γ-lyase (CSE), and the latter two are enzymes within the transsulfuration pathway (TSP). In basal conditions, TSP shunts homocysteine away from the methionine pathway and towards the cysteine biosynthesis pathway, ultimately leading to the production of glutathione (GSH), an important antioxidant molecule in cells [12,13,14].

H_2_S plays a role in cellular energy metabolism through its involvement in mitochondrial respiration, the Krebs cycle, and glycolysis [15,16,17]. H_2_S is a respiratory toxin at high concentrations and inhibits ATP generation of the mitochondrial electron transport chain (ETC) [18,19]. Cellular metabolism regulates the activity of macrophages [20], and H_2_S may be implicated in supporting inflammation-induced metabolic rewiring [21]. Increased H_2_S production via CSE supports the pro-inflammatory response of macrophages that contributes to inflammatory diseases in in vivo models of sepsis [22,23]. However, recent studies utilizing H_2_S donors in similar models of induced sepsis note an opposite and anti-inflammatory role of this molecule [24,25,26,27]. These conflicting results underscore the importance of considering the concentration of H_2_S and its source in determining its function. Here, we review the recent published works covering the role of H_2_S in regulating macrophage activity and its potential role in macrophage-associated diseases.

## 2. The Synthesis of Hydrogen Sulfide in Mammalian Cells

H_2_S is synthesized in mammalian cells predominantly by the TSP enzymes CBS and CSE that link the essential amino acid methionine to the GSH biosynthesis reactions via the metabolic intermediate, homocysteine (Hcy) [28,29]. A recent review by Filipovic et al. (2018) provides an excellent description of the enzymatic reactions involved in the formation of H_2_S, which is not discussed in exhaustive detail here [29]. In addition to TSP enzymes, mercaptopyruvate sulfurtransferase (MST) represents another enzyme responsible for synthesizing H_2_S within cells [30,31]. The first step in the TSP, whereby Hcy is condensed with serine into cystathionine, is carried out by CBS with cofactor pyridoxal 5′-phosphate (PLP) via β-replacement reaction [32]. CSE acts primarily on CBS-derived cystathionine with PLP to generate cysteine [33]. H_2_S is generated in TSP primarily by CSE β-elimination reaction utilizing cysteine, or less efficiently, by CBS β replacement reaction of cysteine with either Hcy or a second mole of cysteine [29]. MST generates H_2_S by converting mercaptopyruvate to pyruvate and transfers sulfur forming a persulfide, from which H_2_S can be released [30]. Recent studies suggest that the role of CSE in Hcy clearance may be more significant than previously appreciated. Macrophages treated with homocysteine and drugs that inhibit CSE causes Hcy-induced oxidative stress and stimulate inflammation and the elevated release of pro-inflammatory cytokines. This suggests that CSE may play a critical role in protecting cells against the harmful effects of Hcy and that its importance in Hcy metabolism and clearance has been underestimated [34].

## 3. The Balancing Act: How Cells Control Cysteine and Hydrogen Sulfide Synthesis through the TSP Regulation

**Figure 1:**
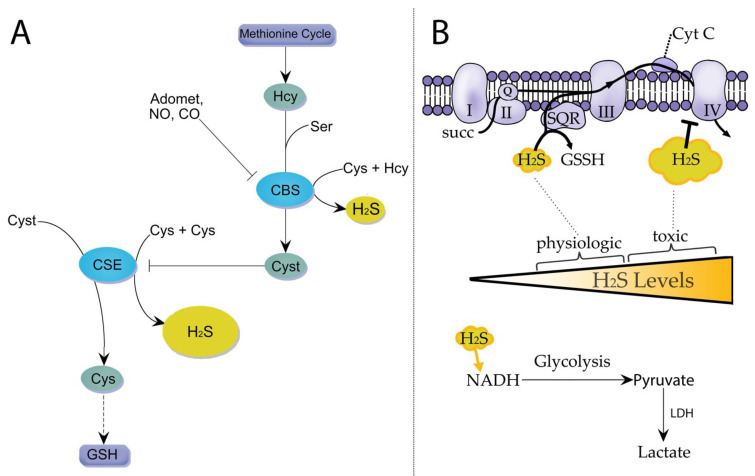
Proposed role of the transsulfuration pathway in producing H_2_S (**A**,**B**). Homocysteine (Hcy) is utilized with serine (Ser) by CBS to produce cystathionine (Cyst). The heme domain within CBS is sensitive to allosteric regulation by ligands (NO, CO, and Adomet), whose concentrations change transiently in response to stimuli. CBS may catalyze H_2_S elimination following condensation of cysteine (Cys) with Hcy. Under basal conditions, Cyst outcompetes Cys utilization by CSE to generate Cys, which contributes to glutathione (GSH) synthesis. Heme-regulated switching of CBS activity or changes in CBS protein levels regulate CSE-derived H_2_S versus cysteine synthesis (Panel **A**). H_2_S exerts a biphasic role upon mitochondrial bioenergetics, and H_2_S concentrations influence electron transport activity. The regulation by H_2_S occurs in an intercompartmental manner whereby H_2_S impacts mitochondrial and cytosolic metabolism (Panel **B**). Serine (Ser), cystathionine B-synthase (CBS), cystathionine y-lyase (CSE), cytochrome C (Cyt C), succinate (succ), glutathione persulfide (GSSH), sulfidequinone oxidoreductase (SQR), lactate dehydrogenase (LDH), hydrogen sulfide (H_2_S).

TSP is highly regulated due to its role in diverting sulfur from the essential amino acid, methionine, to other sulfur metabolites such as cysteine, taurine, GSH, and H_2_S. CSE and CBS are regulated to control the production of H_2_S versus cysteine. The first enzyme in the TSP, CBS, is constitutively expressed in cells, and is reported to be expressed in human primary macrophages and mouse-derived J774A.1 macrophages [35,36,37]. The CBS protein has a heme domain with binding sites for ligands that cause allosteric regulation of CBS’s catalytic activity [29,38,39]. The heme domain exhibits affinity for several small molecules, including S-adenosylmethionine (Adomet) [40,41], as well as the gaseous signaling molecules, NO and CO, each of which inhibits CBS enzymatic activity [38,39]. The co-binding of Adomet can enhance the inhibitory effects of NO and CO on CBS [42,43]. Apart from this posttranslational regulation, CBS protein levels are downregulated in differentiated M1 macrophages of both mouse and human origin following exposure to interferon (IFN)γ and LPS, indicating that inflammation induces rewiring of TSP activity to produce H_2_S [36,37]. The decrease in CBS protein expression or its allosteric inhibition by NO or CO is hypothesized to cause depletion of cystathionine [29], suggesting a mechanism by which the TSP is rewired to decrease cystathionine levels during the inflammatory response in macrophages. Inhibition of CBS activity or a reduction in its protein levels disrupts the biosynthesis of cystathionine, the essential substrate CSE utilizes for cysteine production. Under these conditions, CSE only catalyzes reactions for synthesizing H_2_S, utilizing cellular cysteine pools (Figure 1) [44].

CSE synthesizes cysteine from the essential amino acid methionine via the metabolic intermediate homocysteine [29]. Cysteine derived from TSP contributes to the production of approximately one-half of the intracellular GSH pool in human liver cells [45]. The transcription factor specificity protein (SP)1 maintains physiologic levels of gene transcript *cth*, the gene encoding CSE [46,47]. During inflammatory responses in macrophages, nuclear factor (NF)-κB induces the mRNA expression of CSE, leading to increased protein levels [48] and an increase in H_2_S synthesis [49]. H_2_S induction during inflammation is hypothesized to support signaling during the immune response in macrophages. In summary, the regulation of the TSP is a complex process that involves regulation of protein expression, substrate availability, and posttranslational modification of CBS activities, all of which play a role in determining the pathway’s response to cellular demands for cysteine versus H_2_S synthesis.

## 4. Persulfidation Is a Regulatory Mechanism for Cellular Processes

S-persulfidation by H_2_S or polysulfides targeting cysteine residues on proteins forming persulfide may have a functional relevance in protein function [50,51]. These posttranslational marks exist in cells natively, and the number of persulfidated proteins only increases in cells exposed to H_2_S [51]. There is debate about the primary source of protein persulfide, which are suggested to derive from either sulfide or persulfide formed by H_2_S metabolism. Nonetheless, a majority of reactive persulfides are generated by the oxidizing activities of sulfide quinone oxidoreductase (SQR), a diflavin enzyme localized to the inner-mitochondrial membrane [29]. Persulfides can be formed following oxidation of H_2_S by SQR, forming reactive persulfides that can directly interact with and protect from the damaging effects of reactive oxygen species (ROS) through the formation of several intermediates [29,52,53]. Persulfidation can enhance the activity of antioxidant enzymes. For example, the persulfidation of GSH peroxidase (GPx) reduces lipid peroxidation and enhances antioxidant defense [54]. In addition, endogenous polysulfide production is linked to the regulation of gene transcription by polysulfides. SQR and rhodanese are two enzymes that contribute to this process through the production and transsulfuration of polysulfides. SQR is a major source of polysulfides and is hypothesized to sustain cellular levels of these species [55]. CysSSH and other more reactive persulfides, which are mainly synthesized from cysteine catabolism, serve as signaling molecules that sustain prolonged steady-state levels of several polysulfides in the mitochondria [56,57]. Polysulfide generation, induced by exposure to exogenous or endogenous H_2_S, leads to sustained levels of polysulfides [55], which may induce protective effects. In calcific aortic valve disease (CAVD), the decreased availability of bioactive H_2_S is caused by the increased expression of CSE alongside the up-regulation of SQR-mediated H_2_S metabolism in affected tissues. Notably, treatment with a mitochondria-targeted H_2_S donor has been shown to mitigate inflammation and rescue macrophages from an osteoblastic phenotype switch in this disease context [58]. The activities of some effects previously attributed to H_2_S signaling may instead be a result of polysulfide molecules generated by H_2_S, which react with cysteine residues via protein persulfidation [59]. The main source of intracellular persulfides is H_2_S, produced predominantly by CSE [60]. Studies indicate that pharmacological levels of H_2_S by several types of H_2_S-donor molecules enhance the formation of persulfides [52,59,60]. A recent study investigated the effects of slow-releasing H_2_S and the persulfide donor P* on inducible nitric oxide synthase (iNOS) in chondrocyte-like cells and macrophages. P* effectively reduced iNOS signaling, regardless of H_2_S presence, while sodium hydrosulfide (NaHS), a fast-acting H_2_S donor, did not exhibit a similar effect. Furthermore, polysulfides lowered the expression of transcription factors, C/EBPβ and δ, both of which were induced by the pro-inflammatory cytokine Interleukin (IL)-1β. These findings emphasize the importance of polysulfides as protective signaling molecules and suggest that C/EBPβ/δ could be potential targets for H_2_S and polysulfide-mediated anti-inflammatory signaling in the context of osteoarthritis [61].

## 5. Hydrogen Sulfide and Redox Homeostasis

Cellular components are sensitive to the damaging effects of ROS and require increased protective measures to mitigate ROS-triggered DNA damage, mitochondrial and endoplasmic reticulum (ER) stress, and uncontrolled immune response [13,62,63]. The effect of oxidative stress on NF-κB activation depends on the cell type and context. In response to oxidative stress, NF-κB may induce the expression of several antioxidants [64]. The levels of superoxide dismutase (SOD) [65,66] and GPx [67], among others, are increased via NF-κB to protect cellular components from self-inflicted damage and contribute to a regulated immune response.

As the primary mechanism of antioxidant defense against ROS and electrophiles, GSH serves a crucial role in maintaining cellular redox homeostasis [63]. GSH is synthesized de novo through two ATP-dependent reactions catalyzed by γ-glutamylcysteine synthase and glutathione synthetase. The redox-active thiol (-SH) of cysteine in GSH is oxidized to GSSG when it reduces target molecules and requires NADPH to restore its reduced form [68]. The CSE enzyme plays a crucial role in maintaining the levels of GSH by producing cysteine, the limiting substrate for GSH synthesis [62]. H_2_S also increases GSH levels by enhancing cysteine transporter activity [69,70] and enhances γ-glutamylcysteine synthase activity, which increases γ-glutamylcysteine levels [71]. NF-κB significantly upregulates CSE during inflammatory response in macrophages [48], and CSE indirectly contributes to antioxidant systems by producing cysteine and H_2_S [69,72,73].

H_2_S levels are regulated to support inflammatory responses and can transduce signaling pathways that lead to metabolic rewiring. H_2_S can also interact with various signaling molecules, such as ROS and NO, which modulate macrophage inflammation potentially reducing oxidative stress in specific disease context including cardiovascular diseases [74]. Additionally, H_2_S can directly affect cellular metabolism by inhibiting mitochondrial respiration and promoting glycolysis, which can lead to a rewiring of metabolic pathways [16,75]. H_2_S activity upon mitochondrial bioenergetics is bi-phasic. The electron transport chain couples redox reactions, creating an electrochemical gradient that leads to the creation of ATP. H_2_S stimulates mitochondrial respiration at lower concentrations but inhibits it at higher concentrations [16,76,77]. Elevated H_2_S levels are potent ETC inhibitors via its binding activities upon the metallic center in complex IV [16]. However, at physiologic levels, H_2_S elevates oxygen consumption rates and electron flow through ETC via SQR [16]. Recent evidence challenges the conventional view that H_2_S only exerts toxic effects in cells; in these conditions where the electron transport chain is obstructed, high levels of H_2_S can continue to undergo oxidation by SQR, leading to a redox cycle with fumarate as the terminal electron acceptor at complex II [78].

Alternatively, in the event of inhibition of the ETC by excessive H_2_S, increased glycolytic flux is triggered by increased cytosolic NADH levels [17]. In response, lactate production is elevated through increased lactate dehydrogenase activity, which serves as a counterbalance. This creates a redox-neutral cycle, allowing the cell to continue producing ATP through glycolysis despite ETC inhibition. The cycle relies on the functioning of the electrogenic glutamate–aspartate transporter, which plays a vital role in the malate–aspartate shuttle [17]. This interaction demonstrates how H_2_S may signal to influence cellular energy metabolism, and place increased glucose substrate demands on cells exposed to H_2_S. When mitochondria are under reductive pressure due to an elevated NADH pool, the normally oxidizing Krebs cycle may become reprogrammed. Indeed, in H_2_S-treated colonocytes, the citrate level was increased due to the reversal of the Krebs cycle via the stimulation of glutamine-dependent reductive carboxylation [16]. Overall, the multilevel regulation of TSP enzymes, coupled with mitochondrial SQR demonstrates a remarkable sulfur sensitive redox system in place that regulates numerous cellular systems including cellular metabolism. Since these systems are highly regulated and induced in macrophages during inflammation, future research is required to elucidate these systems in these cells, which may be targeted to reduce disease severity.

## 6. The Therapeutic Potential of Hydrogen Sulfide and Polysulfides

H_2_S is known for its ability to scavenge reactive oxygen and nitrosyl species, although endogenous levels of H_2_S (10–30 nM) are too low for direct free-radical scavenging [79,80], exogenous H_2_S donors, however, may achieve elevated levels. GYY4137, a slow-releasing H_2_S donor, has demonstrated anti-inflammatory effects and protective properties against various conditions, such as septic peritonitis, acute lung injury, and myocardial injury. In M0/M1 macrophages, GYY4137 treatment increased the expression of CD-206 and IL-10 while reducing iNOS and TNF-α expression in M1 macrophages. When combined with IL-4, GYY4137 treatment enhanced M0 macrophage viability and mineralized particle formation in cocultures with MC3T3-E1 osteoblasts [81]. In sepsis studies, GYY4137 notably alleviated septic peritonitis and protected against cecal ligation and puncture (CLP)-induced acute lung injury by decreasing neutrophil infiltration, ameliorating sepsis-induced lung histopathological alterations, and lessening lung injury severity. This protective effect was associated with reduced levels of PDGFRβ, NF-κB, ASC, NLRP3, caspase-1, and Akt proteins in septic mouse lung tissues [26].

GYY4137 is also reported to mitigate ferroptosis and inhibit autophagy activation in macrophage. This compound reduced macrophage infiltration in septic heart tissue and protected against myocardial injury via the NLRP3 inflammasome pathway by decreasing inflammatory response and myocardial ROS production [27,81]. Furthermore, GYY4137 alleviated ferroptosis in septicemia-induced ALI by upregulating GPx and SLC7A11 expression in lung tissue post-CLP. Upon LPS exposure, the expression of mTOR, P62, and Beclin1 increased, while the LC3II/LC3I ratio decreased; however, GYY4137 treatment proved protective by blocking mTOR signaling and inhibiting autophagy activation [25].

**Figure 2:**
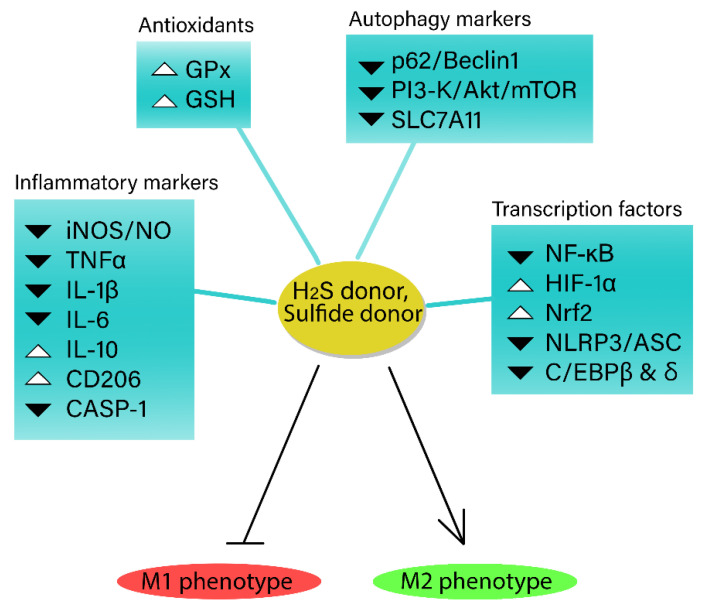
The role of exogenous hydrogen sulfide (H_2_S) in macrophage inflammatory phenotype. H_2_S helps regulate inflammatory responses, while also interacting with pro-inflammatory markers, transcription factors, and proteins to modulate macrophage inflammation. H_2_S donors have demonstrated potential therapeutic benefits in various inflammatory conditions, characterized by inducing anti-inflammatory M2 phenotype in macrophages, highlighting their promise as treatment options.

Recent studies have explored novel H_2_S donor polymers or polysulfide donors, observing similar protective effects. One such novel polymer, which scavenges NO and donates H_2_S, has been shown to decrease ROS levels and pro-inflammatory cytokine production via NF-κB signaling, promoting macrophage M2 polarization and H_2_S release. In a rat model of rheumatoid arthritis, this system significantly reduced synovial inflammation, osteoporosis, and clinical symptoms [82]. Collectively, these findings suggest that H_2_S and its donors hold promise as therapeutic agents for various inflammatory conditions diverting macrophage to the anti-inflammatory phenotype (Figure 2).

## 7. The Interplay between Metabolism and the Immune Response in Macrophages

NF-κB is a central regulator of inflammation, and its activation in macrophages can be triggered by a wide variety of stimuli, including microbial products, pro-inflammatory mediators such as tumor necrosis factor (TNF)-α, IL-1β, and ROS generated during immune response [83]. LPS is a PAMP ligand for the Toll-like receptor (TLR)4 binding domain on the cell membrane, which increases activity of NF-κB. Upon LPS binding, TLR4 dimerizes, and the molecular adapter myeloid differentiation factor 88 (MyD88) is recruited, leading to the activation of the Iκβ kinase (IKK) complex. Activated IKK phosphorylates IκB (inhibitor of NF-κB), leading to its degradation, and subsequent dissociation from NF-κB. This then frees up the p65 subunit of NF-κB to translocate into the nucleus, where it binds to specific DNA sequences and activates the transcription of several target genes involved in immune and inflammatory responses, including TNFα, IL-6, iNOS, and IL-1β [83,84].

Mechanisms are in place to quickly trigger metabolic reprogramming in macrophages upon immune activation. The increased expression of iNOS leads to the inhibition of mitochondrial respiration through the direct competition of NO with oxygen to inhibit cytochrome c oxidase, the terminal enzyme of the ETC [85]. M1 macrophages use arginine to generate NO and citrulline via iNOS, while M2 cells, which do not express iNOS or produce NO, convert arginine to ornithine and urea via type 1 arginase [86]. During M1 macrophage immune activation, argininosuccinate synthase (ASS1) is upregulated, which is an enzyme that increases arginine production to sustain NO levels. Jha et al. (2015) revealed that in M1 macrophages, ASS1-derived NO disrupts mitochondrial oxidative phosphorylation (OXPHOS), requiring macrophages to enhance glycolysis to survive [87]. Glycolysis quickly produces ATP; however, it generates less ATP per glucose molecule than OXPHOS. Nevertheless, glycolysis remains crucial in situations requiring rapid ATP production, such as inflammation.

In response to pro-inflammatory signals, macrophages undergo metabolic reprogramming, enhancing glycolysis to meet energy demands and support activating the immune response [88]. This metabolic reprogramming includes upregulation of the tricarboxylic acid (TCA) cycle and synthesis of arginine and succinate that support immune responses [89]. M1 macrophages increase succinate levels, primarily sourced from glutamine-dependent anaplerosis and the GABA (γ-aminobutyric acid) shunt pathway [89]. During inflammation, the accumulation of succinate in the cytosol, due to its transport from the mitochondria, can lead to the inhibition of prolyl hydroxylase activity and the stabilization of hypoxia inducible factor (HIF)-1α [89,90]. Subsequently, HIF-1α upregulates IL-1β gene transcription [89]. HIF-1α and NF-κB collaborate in macrophages to promote glycolysis and the immune response. NF-κB can lead to the accumulation of HIF-1α by directly binding to its promoter and increasing its transcription [91,92]. In addition, activation of HIF-1α in ex vivo macrophages infected with bacteria is dependent on IKKβ-responsive NF-κB [92]. HIF-1α and NF-κB regulate gene transcription of *slc2a1*, which encodes the primary rate-limiting glucose transporter, Glut1, in macrophages [20,92,93]. Glut1 protein expression is induced to support M1 polarization following LPS stimulation [20,93]. Upon activation, M1 macrophages increase glucose uptake and utilization driving increased ROS by NADPH oxidase activity to combat invading bacteria [94]. To maintain redox balance during M1 polarization, the glycolysis–PPP (pentose–phosphate pathway) axis supports the enhanced production of NADPH used by both NADPH oxidase (NOX) for ROS production, and glutathione reductase that maintains reduced glutathione (GSH) pools to counteract excessive superoxide production [94]. Upregulation of Glut1 is triggered following LPS stimulation and leads to an increase in the production of NOX-derived ROS, driving inflammation [20]. Even in the absence of LPS, transfection to induce the overexpression of Glut1 in RAW264.7 mouse macrophages increased PPP activation and ROS and pro-inflammatory cytokine levels, demonstrating the regulatory role of Glut1 in macrophage immune activation [20].

PI3-K (phosphoinositide 3-kinase) is an enzyme that phosphorylates phosphatidylinositol lipids in the cell membrane, generating second messenger molecules that activate downstream signaling pathways. Akt (protein kinase B) is a serine/threonine kinase and a key downstream effector of PI3-K signaling. Akt is activated by phosphorylation by PI3-K and functions to promote cell survival, growth, and metabolism by phosphorylating and regulating a variety of downstream targets [95,96]. Macrophages stimulated by LPS trigger the activation of PI3-K signaling [97,98], and PI3-K activities are suggested to negatively regulate TLR signaling and prevent overactive immune stimulation of macrophages [98]. Such regulatory activities of PI3-K/Akt signaling are important as they impact the crucial balance between pro-inflammatory and anti-inflammatory responses. The PI3-K/Akt signaling pathway also regulates the expression and trafficking of Glut1, which is vital for the metabolic function of macrophages [99,100]. Akt, a downstream effector of PI3-K, can activate HIF-1α, which induces the expression of Glut1 and enhances glucose metabolism [91,101,102]. The trafficking of Glut1 is an important regulated aspect of glucose uptake that is also stimulated by growth factors, apart from inflammatory signals.

In summary, inflammation activates various pathways, increasing inflammatory cytokines and initiating metabolic reprogramming through enhanced glycolysis. M1 macrophages inhibit mitochondrial respiration, promoting glycolysis. PI3-K/Akt, HIF-1α, and NF-κB work together in macrophages to boost glycolysis and glucose uptake during immune responses. Ultimately, the immune and metabolic responses of macrophages are closely intertwined and essential for effective immunity. The next section will discuss potential crosstalk between H_2_S and cellular energy metabolism.

## 8. The Complex Role of Hydrogen Sulfide in Modulating Cellular Energy Metabolism and Inflammatory Responses in Macrophages

**Figure 3:**
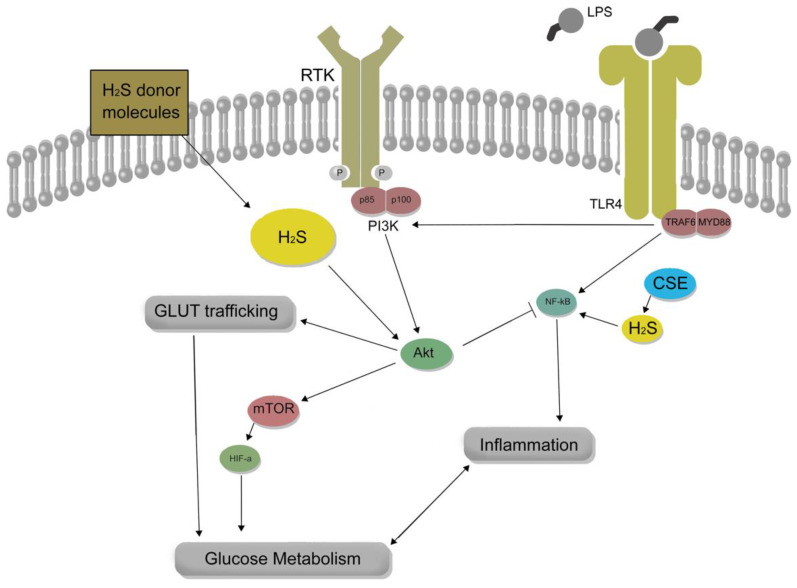
The role of hydrogen sulfide (H_2_S) in macrophage inflammation and glucose metabolism is complex and context-dependent. H_2_S donor molecules can enhance lipopolysaccharide (LPS)-induced Akt activity, which may counteract pro-inflammatory NF-κB activity. Conversely, cystathionine-γ-lyase (CSE)-derived H_2_S supports NF-κB pro-inflammatory activity, leading to the expression of glucose transporter 1 (Glut1) and metabolic reprogramming in macrophages during inflammation. The impact of H_2_S on macrophage inflammation, therefore, depends on factors such as the source, concentration, and context of H_2_S exposure, which can result in either pro- or anti-inflammatory outcomes.

H_2_S plays a key role in regulating cellular energy metabolism and redox homeostasis, which is important for regulating inflammation in macrophages, but its precise role on immune cell inflammatory response is not yet fully understood. Due to the susceptibility of respiration to H_2_S toxicity at high levels, the di-flavin enzyme, SQR, which is localized on the inner mitochondrial membrane, acts as a respiratory shield. H_2_S attacks the disulfide bond in SQR, generating a persulfide charge transfer complex. This outer sulfane sulfur is moved to a thiophilic acceptor, such as GSH, safeguarding ETC function from H_2_S [75,78,103]. H_2_S orchestrates regulatory pathways in cellular bioenergetics, modulating mitochondrial energy production and promoting glycolysis. Previously, it was reported that treatment with the H_2_S-donor molecule, GYY4137, induced the expression of Glut1 by stabilizing HIF-1α under normal oxygen conditions in THP-1 macrophages [104]. This same study also reported that prolonged exposure (24 h) of unstimulated macrophage to high levels of exogenous H_2_S decreased NF-κB and increased Nrf2 activity. We previously reported the role of endogenous H_2_S to support inflammatory Glut1 expression in macrophages via its regulatory role upon NF-κB and Akt activity [21] (Figure 3). Thus, H_2_S plays a vital role in macrophage function by regulating cellular energy metabolism and redox homeostasis, influencing inflammation, and impacting various regulatory pathways that modulate mitochondrial energy production and promote glycolysis.

## 9. Summary and Future Directions

Inflammatory diseases are characterized by excessive inflammation, and macrophages are central players in the pathogenesis of these disorders. It is widely accepted that metabolism is a crucial regulator of macrophage activation and inflammation. Recently, the gasomediator H_2_S has emerged as a potential regulator of cellular energy metabolism, but its precise mechanisms of action in macrophage inflammatory response are not fully understood.

**Figure 4:**
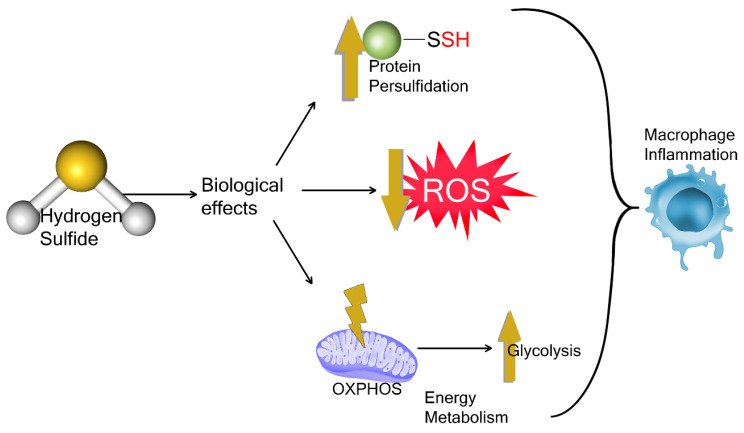
Proposed mechanisms by which H_2_S signals in macrophage. The mechanisms underlying H_2_S signaling in the regulation of macrophage function involve induction of protein persulfidation, cellular redox homeostasis that influences cellular energy metabolism and reactive oxygen species (ROS) formation. H_2_S biological effects are reported to both support and attenuate macrophage inflammation depending upon the source and concentration. OXPHOS (oxidative phosphorylation).

The regulatory role of H_2_S in mitochondrial respiration has been the subject of much discussion regarding its potential to modulate cellular bioenergetics. While the effects of H_2_S on immune cell inflammatory responses remain unclear, recent studies have shed light on the relationship between H_2_S and cellular energy metabolism. H_2_S has a biphasic effect on cellular energy metabolism and potentially participates in inflammatory metabolism rewiring. This intricate interplay highlights how H_2_S may signal to influence cellular energy metabolism and place increased glucose substrate demands on cells exposed to H_2_S. However, this is poorly understood and here we highlight the need for further research to elucidate the specific mechanisms underlying this complex relationship and the implications of these effects in disease states. The interplay between exogenous and endogenous H_2_S and glucose metabolism is an intriguing area of investigation. H_2_S appears to create a favorable redox milieu for glycolysis, suggesting further potential crosstalk. Moreover, it is interesting that H_2_S signals changes to transcription factors and proteins to support inflammatory Glut1 expression in macrophages. In addition, H_2_S mitigates ROS by interacting with signaling molecules, scavenging reactive oxygen and nitrosyl species, and supporting antioxidant systems by producing cysteine and enhancing GSH levels. H_2_S-generated polysulfides contribute to anti-inflammatory signaling and offer protection against ROS, with sulfide donors showing potential as therapeutic agents for various conditions, such as septic peritonitis, acute lung injury, and myocardial injury, by modulating immune responses and reducing inflammation and oxidative stress (Figure 4).

To gain further insight into the impact of the H_2_S on inflammation-induced glucose metabolism, which plays a crucial role in regulating macrophage inflammatory response, future investigations are necessary. Specifically, studies are required to determine the activity of SQR in macrophages during immune responses, which could shed light on the levels of H_2_S metabolic pathways during immune responses. It is possible that SQR is downregulated, simultaneous to TSP rewiring, to increase H_2_S levels, thereby supporting the immune response in macrophages. Further investigations are needed to determine whether the signaling induced by H_2_S is due to molecular H_2_S itself or to the polysulfides generated as a result of H_2_S metabolism. Quantitative analyses are also required to evaluate the extent to which protein function is affected by the post-translational persulfidation. These studies would help shed light on the precise mechanisms underlying the impact of H_2_S on cellular function and provide insight into potential therapeutic targets for diseases associated with dysregulated H_2_S signaling. Further investigation in this area is critical for a more comprehensive understanding of the role of H_2_S in immune cell inflammatory responses.

## 10. Conclusions

In conclusion, inflammatory diseases, in which macrophages play a central role, are marked by excessive inflammation. H_2_S and its donors, such as GYY4137 and novel H_2_S donors and polymers, have demonstrated potential as therapeutic agents for various inflammatory conditions due to their anti-inflammatory effects and protective properties. H_2_S is involved in a range of regulatory pathways, including modulating mitochondrial energy production, promoting glycolysis, and influencing the expression of Glut1 and the activity of NF-κB and Akt. Both GSH and H_2_S play critical roles in maintaining redox homeostasis and supporting inflammatory responses. H_2_S and polysulfides play a role in protein persulfidation, which can have functional relevance in protein function and protect against ROS. Additionally, recent studies suggest that polysulfides are critical signaling molecules, with potential therapeutic implications in inflammatory diseases. Despite its potential as a therapeutic target for reducing disease severity, the precise mechanisms of H_2_S in macrophage inflammatory response and its complex relationship with cellular energy metabolism remain unclarified. Future research is needed to elucidate these mechanisms, their implications in disease states, and the potential therapeutic targets they may offer.

## References

[1] Martinez F.O., Gordon S. (2014). The M1 and M2 Paradigm of Macrophage Activation: Time for Reassessment. F1000Prime Rep..

[2] Herbst S., Schaible U.E., Schneider B.E. (2011). Interferon Gamma Activated Macrophages Kill Mycobacteria by Nitric Oxide Induced Apoptosis. PLoS ONE.

[3] Piedrafita D., Parsons J.C., Sandeman R.M., Wood P.R., Estuningsih S.E., Partoutomo S., Spithill T.W. (2001). Antibody-Dependent Cell-Mediated Cytotoxicity to Newly Excysted Juvenile Fasciola Hepatica in Vitro Is Mediated by Reactive Nitrogen Intermediates. Parasite Immunol..

[4] Thomas G.R., McCrossan M., Selkirk M.E. (1997). Cytostatic and Cytotoxic Effects of Activated Macrophages and Nitric Oxide Donors on Brugia Malayi. Infect. Immun..

[5] Lavin Y., Mortha A., Rahman A., Merad M. (2015). Regulation of Macrophage Development and Function in Peripheral Tissues. Nat. Rev. Immunol..

[6] Sun H.-J., Wu Z.-Y., Nie X.-W., Bian J.-S. (2021). Role of Hydrogen Sulfide and Polysulfides in Neurological Diseases: Focus on Protein S-Persulfidation. Curr. Neuropharmacol..

[7] Wang X.-Q., Congyi W., Sun F., Luo J., Yue T., Wang F., Yang C., Zhang S. (2020). The Hydrogen Sulfide Signaling in Macrophages: A Foe or Friend?. Authorea Prepr..

[8] Bhatia M., Gaddam R.R. (2021). Hydrogen Sulfide in Inflammation: A Novel Mediator and Therapeutic Target. Antioxid Redox Signal.

[9] Fagone P., Mazzon E., Bramanti P., Bendtzen K., Nicoletti F. (2018). Gasotransmitters and the Immune System: Mode of Action and Novel Therapeutic Targets. Eur. J. Pharmacol..

[10] Palmieri E.M., McGinity C., Wink D.A., McVicar D.W. (2020). Nitric Oxide in Macrophage Immunometabolism: Hiding in Plain Sight. Metabolites.

[11] Morse D., Choi A.M.K. (2002). Heme Oxygenase-1: The “Emerging Molecule” Has Arrived. Am. J. Respir. Cell Mol. Biol..

[12] Paul B.D., Snyder S.H. (2018). Gasotransmitter Hydrogen Sulfide Signaling in Neuronal Health and Disease. Biochem. Pharmacol..

[13] McBean G.J., Aslan M., Griffiths H.R., Torrão R.C. (2015). Thiol Redox Homeostasis in Neurodegenerative Disease. Redox Biol..

[14] Sbodio J.I., Snyder S.H., Paul B.D. (2019). Regulators of the Transsulfuration Pathway. Br. J. Pharmacol..

[15] Carballal S., Vitvitsky V., Kumar R., Hanna D.A., Libiad M., Gupta A., Jones J.W., Banerjee R. (2021). Hydrogen Sulfide Stimulates Lipid Biogenesis from Glutamine That Is Dependent on the Mitochondrial NAD(P)H Pool. J. Biol. Chem..

[16] Libiad M., Vitvitsky V., Bostelaar T., Bak D.W., Lee H.J., Sakamoto N., Fearon E., Lyssiotis C.A., Weerapana E., Banerjee R. (2019). Hydrogen Sulfide Perturbs Mitochondrial Bioenergetics and Triggers Metabolic Reprogramming in Colon Cells. J. Biol. Chem..

[17] Vitvitsky V., Kumar R., Libiad M., Maebius A., Landry A.P., Banerjee R. (2021). The Mitochondrial NADH Pool Is Involved in Hydrogen Sulfide Signaling and Stimulation of Aerobic Glycolysis. J. Biol. Chem..

[18] Fu M., Zhang W., Wu L., Yang G., Li H., Wang R. (2012). Hydrogen Sulfide (H2S) Metabolism in Mitochondria and Its Regulatory Role in Energy Production. Proc. Natl. Acad. Sci. USA.

[19] Sen U., Pushpakumar S.B., Amin M.A., Tyagi S.C. (2014). Homocysteine in Renovascular Complications: Hydrogen Sulfide Is a Modulator and Plausible Anaerobic ATP Generator. Nitric Oxide.

[20] Freemerman A.J., Johnson A.R., Sacks G.N., Milner J.J., Kirk E.L., Troester M.A., Macintyre A.N., Goraksha-Hicks P., Rathmell J.C., Makowski L. (2014). Metabolic Reprogramming of Macrophages: Glucose Transporter 1 (GLUT1)-Mediated Glucose Metabolism Drives a Proinflammatory Phenotype. J. Biol. Chem..

[21] Cornwell A., Fedotova S., Cowan S., Badiei A. (2022). Cystathionine γ-Lyase and Hydrogen Sulfide Modulates Glucose Transporter Glut1 Expression via NF-ΚB and PI3k/Akt in Macrophages during Inflammation. PLoS ONE.

[22] Badiei A., Gieseg S., Davies S., Othman M.I., Bhatia M. (2015). LPS Up-Regulates Cystathionine γ -Lyase Gene Expression in Primary Human Macrophages via NF-ΚB/ERK Pathway. Inflamm. Allergy Drug Targets.

[23] Gaddam R.R., Fraser R., Badiei A., Chambers S., Cogger V.C., le Couteur D.G., Ishii I., Bhatia M. (2016). Cystathionine-Gamma-Lyase Gene Deletion Protects Mice against Inflammation and Liver Sieve Injury Following Polymicrobial Sepsis. PLoS ONE.

[24] Chen Y.H., Teng X., Hu Z.J., Tian D.Y., Jin S., Wu Y.M. (2021). Hydrogen Sulfide Attenuated Sepsis-Induced Myocardial Dysfunction Through TLR4 Pathway and Endoplasmic Reticulum Stress. Front. Physiol..

[25] Li J., Li M., Li L., Ma J., Yao C., Yao S. (2022). Hydrogen Sulfide Attenuates Ferroptosis and Stimulates Autophagy by Blocking MTOR Signaling in Sepsis-Induced Acute Lung Injury. Mol. Immunol..

[26] Li J., Ma J., Li M., Tao J., Chen J., Yao C., Yao S. (2021). GYY4137 Alleviates Sepsis-Induced Acute Lung Injury in Mice by Inhibiting the PDGFRβ/Akt/NF-ΚB/NLRP3 Pathway. Life Sci..

[27] Zhou T., Qian H., Zheng N., Lu Q., Han Y. (2022). GYY4137 Ameliorates Sepsis-Induced Cardiomyopathy via NLRP3 Pathway. Biochim. Biophys Acta Mol. Basis. Dis..

[28] Beatty P.W., Reed D.J. (1980). Involvement of the Cystathionine Pathway in the Biosynthesis of Glutathione by Isolated Rat Hepatocytes. Arch. Biochem. Biophys..

[29] Filipovic M.R., Zivanovic J., Alvarez B., Banerjee R. (2018). Chemical Biology of H2S Signaling through Persulfidation. Chem. Rev..

[30] Nagahara N., Ito T., Kitamura H., Nishino T. (1998). Tissue and Subcellular Distribution of Mercaptopyruvate Sulfurtransferase in the Rat: Confocal Laser Fluorescence and Immunoelectron Microscopic Studies Combined with Biochemical Analysis. Histochem. Cell Biol..

[31] Mikami Y., Shibuya N., Kimura Y., Nagahara N., Yamada M., Kimura H. (2011). Hydrogen Sulfide Protects the Retina from Light-Induced Degeneration by the Modulation of Ca2+ Influx. J. Biol. Chem..

[32] Chen X., Jhee K.H., Kruger W.D. (2004). Production of the Neuromodulator H2S by Cystathionine Beta-Synthase via the Condensation of Cysteine and Homocysteine. J. Biol. Chem..

[33] Singh S., Padovani D., Leslie R.A., Chiku T., Banerjee R. (2009). Relative Contributions of Cystathionine Beta-Synthase and Gamma-Cystathionase to H2S Biogenesis via Alternative Trans-Sulfuration Reactions. J. Biol. Chem..

[34] Li J.J., Li Q., Du H.P., Wang Y.L., You S.J., Wang F., Xu X.S., Cheng J., Cao Y.J., Liu C.F. (2015). Homocysteine Triggers Inflammatory Responses in Macrophages through Inhibiting CSE-H2S Signaling via DNA Hypermethylation of CSE Promoter. Int. J. Mol. Sci..

[35] Bronowicka-Adamska P., Hutsch T., Gawrys-Kopczynska M., Maksymiuk K., Wróbel M. (2019). Hydrogen Sulfide Formation in Experimental Model of Acute Pancreatitis. Acta Biochim. Pol..

[36] Bronowicka-Adamska P., Jurkowska H., Gawda A., Skalska P., Nazimek K., Marcinkiewicz J., Wróbel M. (2019). Expression and Activity of Hydrogen Sulfide Generating Enzymes in Murine Macrophages Stimulated with Lipopolysaccharide and Interferon-γ. Mol. Biol. Rep..

[37] Garg S., Vitvitsky V., Gendelman H.E., Banerjee R. (2006). Monocyte Differentiation, Activation, and Mycobacterial Killing Are Linked to Transsulfuration-Dependent Redox Metabolism. J. Biol. Chem..

[38] Taoka S., Lepore B.W., Kabil Ö., Ojha S., Ringe D., Banerjee R. (2002). Human Cystathionine Beta-Synthase Is a Heme Sensor Protein. Evidence That the Redox Sensor Is Heme and Not the Vicinal Cysteines in the CXXC Motif Seen in the Crystal Structure of the Truncated Enzyme. Biochemistry.

[39] Taoka S., Banerjee R. (2001). Characterization of NO Binding to Human Cystathionine β-Synthase: Possible Implications of the Effects of CO and NO Binding to the Human Enzyme. J. Inorg. Biochem..

[40] Ereño-Orbea J., Majtan T., Oyenarte I., Kraus J.P., Martínez-Cruza L.A. (2013). Structural Basis of Regulation and Oligomerization of Human Cystathionine β-Synthase, the Central Enzyme of Transsulfuration. Proc. Natl. Acad. Sci. USA.

[41] Scott J.W., Hawley S.A., Green K.A., Anis M., Stewart G., Scullion G.A., Norman D.G., Hardie D.G. (2004). CBS Domains Form Energy-Sensing Modules Whose Binding of Adenosine Ligands Is Disrupted by Disease Mutations. J. Clin. Investig..

[42] Vicente J.B., Colaço H.G., Sarti P., Leandro P., Giuffrè A. (2016). S-Adenosyl-l-Methionine Modulates CO and NO• Binding to the Human H_2_S-Generating Enzyme Cystathionine β-Synthase. J. Biol. Chem..

[43] Vicente J.B., Malagrinò F., Arese M., Forte E., Sarti P., Giuffrè A. (2016). Bioenergetic Relevance of Hydrogen Sulfide and the Interplay between Gasotransmitters at Human Cystathionine β-Synthase. Biochim. Biophys. Acta.

[44] Kabil O., Yadav V., Banerjee R. (2016). Heme-Dependent Metabolite Switching Regulates H2S Synthesis in Response to Endoplasmic Reticulum (ER) Stress. J. Biol. Chem..

[45] Mosharov E., Cranford M.R., Banerjee R. (2000). The Quantitatively Important Relationship between Homocysteine Metabolism and Glutathione Synthesis by the Transsulfuration Pathway and Its Regulation by Redox Changes. Biochemistry.

[46] Zhang L., Yang G., Tang G., Wu L., Wang R. (2011). Rat Pancreatic Level of Cystathionine γ-Lyase Is Regulated by Glucose Level via Specificity Protein 1 (SP1) Phosphorylation. Diabetologia.

[47] Yang G., Pei Y., Teng H., Cao Q., Wang R. (2011). Specificity Protein-1 as a Critical Regulator of Human Cystathionine Gamma-Lyase in Smooth Muscle Cells. J. Biol. Chem..

[48] Badiei A., Muniraj N., Chambers S., Bhatia M. (2014). Inhibition of Hydrogen Sulfide Production by Gene Silencing Attenuates Inflammatory Activity by Downregulation of NF-ΚB and MAP Kinase Activity in LPS-Activated RAW 264.7 Cells. Biomed Res. Int..

[49] Zheng Y., Luo N., Mu D., Jiang P., Liu R., Sun H., Xiong S., Liu X., Wang L., Chu Y. (2013). Lipopolysaccharide Regulates Biosynthesis of Cystathionine γ-Lyase and Hydrogen Sulfide through Toll-like Receptor-4/P38 and Toll-like Receptor-4/NF-ΚB Pathways in Macrophages. Vitr. Cell Dev. Biol. Anim..

[50] Mishanina T.V., Libiad M., Banerjee R. (2015). Biogenesis of Reactive Sulfur Species for Signaling by Hydrogen Sulfide Oxidation Pathways. Nat. Chem. Biol..

[51] Gao X.H., Krokowski D., Guan B.J., Bederman I., Majumder M., Parisien M., Diatchenko L., Kabil O., Willard B., Banerjee R. (2015). Quantitative H2S-Mediated Protein Sulfhydration Reveals Metabolic Reprogramming during the Integrated Stress Response. Elife.

[52] Greiner R., Pálinkás Z., Bäsell K., Becher D., Antelmann H., Nagy P., Dick T.P. (2013). Polysulfides Link H2S to Protein Thiol Oxidation. Antioxid Redox Signal.

[53] Kimura Y., Mikami Y., Osumi K., Tsugane M., Oka J.I., Kimura H. (2013). Polysulfides Are Possible H2S-Derived Signaling Molecules in Rat Brain. FASEB J..

[54] Zhao Z.Z., Wang Z., Li G.H., Wang R., Tan J.M., Cao X., Suo R., Jiang Z.S. (2011). Hydrogen Sulfide Inhibits Macrophage-Derived Foam Cell Formation. Exp. Biol. Med..

[55] Shimizu T., Ida T., Antelo G.T., Ihara Y., Fakhoury J.N., Masuda S., Giedroc D.P., Akaike T., Capdevila D.A., Masuda T. (2023). Polysulfide Metabolizing Enzymes Influence SqrR-Mediated Sulfide-Induced Transcription by Impacting Intracellular Polysulfide Dynamics. PNAS Nexus.

[56] Akaike T., Ida T., Wei F.Y., Nishida M., Kumagai Y., Alam M.M., Ihara H., Sawa T., Matsunaga T., Kasamatsu S. (2017). Cysteinyl-TRNA Synthetase Governs Cysteine Polysulfidation and Mitochondrial Bioenergetics. Nat. Commun..

[57] Ida T., Sawa T., Ihara H., Tsuchiya Y., Watanabe Y., Kumagai Y., Suematsu M., Motohashi H., Fujii S., Matsunaga T. (2014). Reactive Cysteine Persulfides and S-Polythiolation Regulate Oxidative Stress and Redox Signaling. Proc. Natl. Acad. Sci. USA.

[58] Combi Z., Potor L., Nagy P., Sikura K.É., Ditrói T., Jurányi E.P., Galambos K., Szerafin T., Gergely P., Whiteman M. (2023). Hydrogen Sulfide as an Anti-Calcification Stratagem in Human Aortic Valve: Altered Biogenesis and Mitochondrial Metabolism of H2S Lead to H2S Deficiency in Calcific Aortic Valve Disease. Redox Biol..

[59] Kimura H. (2020). Signalling by Hydrogen Sulfide and Polysulfides via Protein S-Sulfuration. Br. J. Pharmacol..

[60] Zivanovic J., Kouroussis E., Kohl J.B., Adhikari B., Bursac B., Schott-Roux S., Petrovic D., Miljkovic J.L., Thomas-Lopez D., Jung Y. (2019). Selective Persulfide Detection Reveals Evolutionarily Conserved Anti-Aging Effects of S-Sulfhydration. Cell Metab..

[61] Trummer M., Galardon E., Mayer B., Steiner G., Stamm T., Kloesch B. (2022). Polysulfides Derived from the Hydrogen Sulfide and Persulfide Donor P* Inhibit IL-1β-Mediated Inducible Nitric Oxide Synthase Signaling in ATDC5 Cells: Are CCAAT/Enhancer-Binding Proteins β and δ Involved in the Anti-Inflammatory Effects of Hydrogen Sulfide and Polysulfides?. Nitric. Oxide.

[62] Lee Z.W., Low Y.L., Huang S., Wang T., Deng L.W. (2014). The Cystathionine γ-Lyase/Hydrogen Sulfide System Maintains Cellular Glutathione Status. Biochem. J..

[63] Ribas V., García-Ruiz C., Fernández-Checa J.C. (2014). Glutathione and Mitochondria. Front. Pharmacol..

[64] Lingappan K. (2018). NF-ΚB in Oxidative Stress. Curr. Opin. Toxicol..

[65] Das K.C., Lewis-Molock Y., White C.W. (1995). Activation of NF-Kappa B and Elevation of MnSOD Gene Expression by Thiol Reducing Agents in Lung Adenocarcinoma (A549) Cells. Am. J. Physiol..

[66] Rojo A.I., Salinas M., Martín D., Perona R., Cuadrado A. (2004). Regulation of Cu/Zn-Superoxide Dismutase Expression via the Phosphatidylinositol 3 Kinase/Akt Pathway and Nuclear Factor-KappaB. J. Neurosci..

[67] Schreiber J., Jenner R.G., Murray H.L., Gerber G.K., Gifford D.K., Young R.A. (2006). Coordinated Binding of NF-KappaB Family Members in the Response of Human Cells to Lipopolysaccharide. Proc. Natl. Acad. Sci. USA.

[68] Pompella A., Visvikis A., Paolicchi A., de Tata V., Casini A.F. (2003). The Changing Faces of Glutathione, a Cellular Protagonist. Biochem. Pharmacol..

[69] Kimura Y., Goto Y.I., Kimura H. (2010). Hydrogen Sulfide Increases Glutathione Production and Suppresses Oxidative Stress in Mitochondria. Antioxid Redox Signal.

[70] Kimura H. (2015). Signaling Molecules: Hydrogen Sulfide and Polysulfide. Antioxid Redox Signal.

[71] Kimura Y., Kimura H. (2004). Hydrogen Sulfide Protects Neurons from Oxidative Stress. FASEB J..

[72] Sun F., Luo J.H., Yue T.T., Wang F.X., Yang C.L., Zhang S., Wang X.Q., Wang C.Y. (2021). The Role of Hydrogen Sulphide Signalling in Macrophage Activation. Immunology.

[73] Sun W.H., Liu F., Chen Y., Zhu Y.C. (2012). Hydrogen Sulfide Decreases the Levels of ROS by Inhibiting Mitochondrial Complex IV and Increasing SOD Activities in Cardiomyocytes under Ischemia/Reperfusion. Biochem. Biophys. Res. Commun..

[74] Zhang H., Du J., Huang Y., Tang C., Jin H. (2023). Hydrogen Sulfide Regulates Macrophage Function in Cardiovascular Diseases. Antioxid Redox Signal.

[75] Vitvitsky V., Kabil O., Banerjee R. (2012). High Turnover Rates for Hydrogen Sulfide Allow for Rapid Regulation of Its Tissue Concentrations. Antioxid Redox Signal.

[76] Goubern M., Andriamihaja M., Nübel T., Blachier F., Bouillaud F. (2007). Sulfide, the First Inorganic Substrate for Human Cells. FASEB J..

[77] Lagoutte E., Mimoun S., Andriamihaja M., Chaumontet C., Blachier F., Bouillaud F. (2010). Oxidation of Hydrogen Sulfide Remains a Priority in Mammalian Cells and Causes Reverse Electron Transfer in Colonocytes. Biochim. Biophys. Acta.

[78] Kumar R., Landry A.P., Guha A., Vitvitsky V., Lee H.J., Seike K., Reddy P., Lyssiotis C.A., Banerjee R. (2021). A Redox Cycle with Complex II Promotes Sulfide Quinone Oxidoreductase Dependent H_2_S Oxidation. bioRxiv.

[79] Furne J., Saeed A., Levitt M.D. (2008). Whole Tissue Hydrogen Sulfide Concentrations Are Orders of Magnitude Lower than Presently Accepted Values. Am. J. Physiol. Regul. Integr. Comp. Physiol..

[80] Levitt M.D., Abdel-Rehim M.S., Furne J. (2011). Free and Acid-Labile Hydrogen Sulfide Concentrations in Mouse Tissues: Anomalously High Free Hydrogen Sulfide in Aortic Tissue. Antioxid Redox Signal.

[81] Zhou T., Liu W., Lai H., Liu Y., Su W., Xu Z. (2023). Hydrogen Sulfide Promotes Osteogenesis by Modulating Macrophage Polarization. Int. Immunopharmacol..

[82] Geng W., Liu X., Tao B., He Y., Li K., Gao P., Feng Q., Zhao P., Luo Z., Cai K. (2023). Nitric Oxide Scavenging and Hydrogen Sulfide Production Synergistically Treat Rheumatoid Arthritis. Adv. Healthc. Mater.

[83] Mulero M.C., Huxford T., Ghosh G. (2019). NF-ΚB, IκB, and IKK: Integral Components of Immune System Signaling. Adv. Exp. Med. Biol..

[84] Lu Y.C., Yeh W.C., Ohashi P.S. (2008). LPS/TLR4 Signal Transduction Pathway. Cytokine.

[85] Everts B., Amiel E., van der Windt G.J.W., Freitas T.C., Chott R., Yarasheski K.E., Pearce E.L., Pearce E.J. (2012). Commitment to Glycolysis Sustains Survival of NO-Producing Inflammatory Dendritic Cells. Blood.

[86] Shearer J.D., Richards J.R., Mills C.D., Caldwell M.D. (1997). Differential Regulation of Macrophage Arginine Metabolism: A Proposed Role in Wound Healing. Am. J. Physiol..

[87] Jha A.K., Huang S.C.C., Sergushichev A., Lampropoulou V., Ivanova Y., Loginicheva E., Chmielewski K., Stewart K.M., Ashall J., Everts B. (2015). Network Integration of Parallel Metabolic and Transcriptional Data Reveals Metabolic Modules That Regulate Macrophage Polarization. Immunity.

[88] Kelly B., O’Neill L.A.J. (2015). Metabolic Reprogramming in Macrophages and Dendritic Cells in Innate Immunity. Cell Res..

[89] Tannahill G.M., Curtis A.M., Adamik J., Palsson-Mcdermott E.M., McGettrick A.F., Goel G., Frezza C., Bernard N.J., Kelly B., Foley N.H. (2013). Succinate Is an Inflammatory Signal That Induces IL-1β through HIF-1α. Nature.

[90] Selak M.A., Armour S.M., MacKenzie E.D., Boulahbel H., Watson D.G., Mansfield K.D., Pan Y., Simon M.C., Thompson C.B., Gottlieb E. (2005). Succinate Links TCA Cycle Dysfunction to Oncogenesis by Inhibiting HIF-α Prolyl Hydroxylase. Cancer Cell.

[91] Cramer T., Yamanishi Y., Clausen B.E., Förster I., Pawlinski R., Mackman N., Haase V.H., Jaenisch R., Corr M., Nizet V. (2003). HIF-1α Is Essential for Myeloid Cell-Mediated Inflammation. Cell.

[92] Rius J., Guma M., Schachtrup C., Akassoglou K., Zinkernagel A.S., Nizet V., Johnson R.S., Haddad G.G., Karin M. (2008). NF-KappaB Links Innate Immunity to the Hypoxic Response through Transcriptional Regulation of HIF-1alpha. Nature.

[93] Obaid M., Udden S.M.N., Alluri P., Mandal S.S. (2021). LncRNA HOTAIR Regulates Glucose Transporter Glut1 Expression and Glucose Uptake in Macrophages during Inflammation. Sci. Rep..

[94] Sheppard F.R., Kelher M.R., Moore E.E., McLaughlin N.J.D., Banerjee A., Silliman C.C. (2005). Structural Organization of the Neutrophil NADPH Oxidase: Phosphorylation and Translocation during Priming and Activation. J. Leukoc. Biol..

[95] Kane L.P., Shapiro V.S., Stokoe D., Weiss A. (1999). Induction of NF-KappaB by the Akt/PKB Kinase. Curr. Biol..

[96] Vergadi E., Ieronymaki E., Lyroni K., Vaporidi K., Tsatsanis C. (2017). Akt Signaling Pathway in Macrophage Activation and M1/M2 Polarization. J. Immunol..

[97] Luyendyk J.P., Schabbauer G.A., Tencati M., Holscher T., Pawlinski R., Mackman N. (2008). Genetic Analysis of the Role of the PI3K-Akt Pathway in Lipopolysaccharide-Induced Cytokine and Tissue Factor Gene Expression in Monocytes/Macrophages. J. Immunol..

[98] Fukao T., Koyasu S. (2003). PI3K and Negative Regulation of TLR Signaling. Trends Immunol..

[99] Cheng S.C., Quintin J., Cramer R.A., Shepardson K.M., Saeed S., Kumar V., Giamarellos-Bourboulis E.J., Martens J.H.A., Rao N.A., Aghajanirefah A. (2014). MTOR- and HIF-1α-Mediated Aerobic Glycolysis as Metabolic Basis for Trained Immunity. Science.

[100] Covarrubias A.J., Aksoylar H.I., Horng T. (2015). Control of Macrophage Metabolism and Activation by MTOR and Akt Signaling. Semin. Immunol..

[101] Joshi S., Singh A.R., Zulcic M., Durden D.L. (2014). A Macrophage-Dominant PI3K Isoform Controls Hypoxia-Induced HIF1α and HIF2α Stability and Tumor Growth, Angiogenesis, and Metastasis. Mol. Cancer Res..

[102] Li C., Wang Y., Li Y., Yu Q., Jin X., Wang X., Jia A., Hu Y., Han L., Wang J. (2018). HIF1α-Dependent Glycolysis Promotes Macrophage Functional Activities in Protecting against Bacterial and Fungal Infection. Sci. Rep..

[103] Landry A.P., Ballou D.P., Banerjee R. (2021). Hydrogen Sulfide Oxidation by Sulfide Quinone Oxidoreductase. Chembiochem.

[104] Lohninger L., Tomasova L., Praschberger M., Hintersteininger M., Erker T., Gmeiner B.M.K., Laggner H. (2015). Hydrogen Sulphide Induces HIF-1α and Nrf2 in THP-1 Macrophages. Biochimie.

